# Advanced Materials Design for Adsorption of Toxic Substances in Cigarette Smoke

**DOI:** 10.1002/advs.202301834

**Published:** 2023-05-21

**Authors:** Ting Zeng, Yanxia Liu, Yingfang Jiang, Lan Zhang, Yagang Zhang, Lin Zhao, Xiaoli Jiang, Qiang Zhang

**Affiliations:** ^1^ School of Materials and Energy University of Electronic Science and Technology of China Chengdu 611731 China; ^2^ Research Center Chengdu Medical College Chengdu 610500 China; ^3^ Univ Lyon CNRS INSA‐Lyon Université Claude Bernard Lyon 1 CETHIL UMR5008 Villeurbanne F‐69621 France; ^4^ Department of Chemistry Washington State University Pullman WA 99164 USA

**Keywords:** cigarette smoke, toxic compounds, adsorption materials, cigarette filters, carcinogens controls

## Abstract

Cigarettes, despite being economically important legal consumer products, are highly addictive and harmful, particularly to the respiratory system. Tobacco smoke is a complex mixture containing over 7000 chemical compounds, 86 of which are identified to have “sufficient evidence of carcinogenicity” in either animal or human tests. Thus, tobacco smoke poses a significant health risk to humans. This article focuses on materials that help reduce the levels of major carcinogens in cigarette smoke; these include nicotine, polycyclic aromatic hydrocarbons, tobacco‐specific nitrosamines, hydrogen cyanide, carbon monoxide, and formaldehyde. Specifically, the research progress on adsorption effects and mechanisms of advanced materials such as cellulose, zeolite, activated carbon, graphene, and molecularly imprinted polymers are highlighted. The future trends and prospects in this field are also discussed. Notably, with advancements in supramolecular chemistry and materials engineering, the design of functionally oriented materials has become increasingly multidisciplinary. Certainly, several advanced materials can play a critical role in reducing the harmful effects of cigarette smoke. This review aims to serve as an insightful reference for the design of hybrid and functionally oriented advanced materials.

## Graphical abstract

For many years, CA is used as a cigarette smoke filter, but there are recent developments in absorbent materials such as zeolite and its derivatives, AC composites, modified graphene, MIPs, various composites, and hybrid materials. The question is how to design the next generation of highly selective, emerging advanced materials that can protect smokers from the harmful components of cigarette smoke.

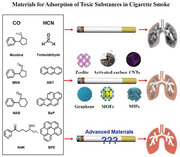

## 1 Introduction

### 1.1 Tobacco Industry and Human Health

**Figure 1:**
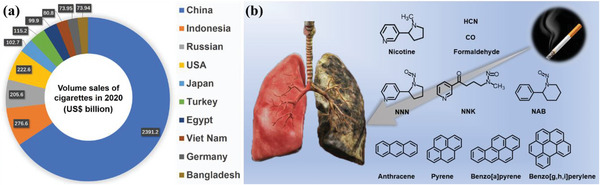
a) TOP 10 cigarette markets in 2020; b) toxic substances in cigarette smoke.^[^ ^2^ ^]^

Tobacco is a widely cultivated cash crop and one of the world's largest economic crops. The six largest tobacco producers include China, India, Brazil, the United States, Indonesia, and Malawi. Currently, > 120 countries cultivate tobacco. Although the smoking trend appears to be declining in most countries, the absolute number of smokers remains high, fueled by nicotine addiction and population growth. Tobacco consumption poses significant risks to human health. Despite the implementation of tobacco control measures, the number of smokers, including young people, remains a cause of concern. Although tobacco sales have decreased slightly in specific regions owing to global tobacco control efforts, sales continue to increase in most areas. **Figure** **1**a highlights the top ten cigarette markets by sales volume in 2020.

Approximately 20% of the world's estimated 1.1 billion smokers reside in high‐income countries. Tobacco use causes almost eight million deaths annually, with 1.2 million from secondhand smoke, making it a primary cause of premature mortality globally. Smoking, which includes the use of cigarettes and electronic cigarettes (e‐cigarettes), is associated with various health risks such as respiratory and cardiovascular diseases, diabetes, and cancer. Smoking can reduce life expectancy by at least ten years. Tobacco smoke has emerged as a significant health hazard to humans with far‐reaching consequences for individuals, families, and society.^[^ ^1^ ^]^ The detrimental effects of smoking can be ascribed to the numerous toxic substances present in cigarette smoke, including nicotine, polycyclic aromatic hydrocarbons (PAHs), tobacco‐specific nitrosamines (TSNA), hydrogen cyanide (HCN), carbon monoxide (CO), and formaldehyde (Figure 1b). These chemicals have been linked to various health hazards including cancer, respiratory illnesses, and cardiovascular diseases. Toxic substances present in cigarette smoke are the major causes of the high morbidity and mortality rates.^[^ ^2^ ^]^ Cigarette smoking is a major factor responsible for pulmonary health issues such as pulmonary fibrosis, asthma, and chronic obstructive pulmonary disease. Lung cancer cases and mortality rates have significantly increased worldwide over the past 50 years, with an annual increase of ≈3% in new lung cancer cases. Cigarette smoking has been proven to be associated with cancer in various organs, including the liver, stomach, larynx, esophagus, pancreas, and urinary bladder. Epidemiological and experimental studies have shown that smoking‐induced alterations in the central nervous system, primarily centered on inflammatory and oxidative stress pathways, can have structural and functional effects. Tobacco smoking is associated with a higher risk of various neuropsychiatric conditions, including dementia/cognitive decline, schizophrenia/psychosis, depression, and suicidal behavior.^[^ ^3^ ^]^ Li et al.^[^ ^4^ ^]^ reported that smoking is a significant risk factor for intestinal disorders such as Crohn's disease and peptic ulcers. Additionally, smoking has negative effects on reproductive health, increasing the incidence of low birth weight and premature births.^[^ ^5^ ^]^ These adverse outcomes can have far‐reaching consequences for individuals, families, and society at large, making it essential to raise awareness of the health risks associated with smoking and implement effective tobacco control policies.

### 1.2 Harm Reduction Approaches in Tobacco Industry

The World Health Organization has recommended six MPOWER measures to combat the global tobacco epidemic. Various measures can be adopted to mitigate the negative effects of tobacco use. These include closely monitoring tobacco usage, implementing preventive measures, providing support for those who wish to quit smoking, educating people about the high risks associated with smoking, increasing tobacco taxes, and implementing law enforcement rules on tobacco promotion and advertising. While many countries have made progress in curbing smoking, some countries are yet to address emerging nicotine and tobacco products or develop effective regulations. Currently, the primary methods for reducing harm to the tobacco industry include tobacco cultivation, leaf processing, cigarette processing, and new cigarette filters.

#### 1.2.1 Tobacco Cultivation

Tobacco leaves, a raw material for cigarettes, produce tar and other harmful components when burned. Agronomic measures can be implemented to promote desired nicotine concentrations. In breeding tobacco varieties, it is necessary to cultivate varieties with low nicotine and tar contents but sufficient aroma, as well as other varieties that meet specific requirements. Scientists have conducted studies on the synthesis of tobacco alkaloids using molecular genetic approaches. During tobacco cultivation, nicotine concentrations can be affected by applying nitrogen fertilizers, adjusting planting density, inhibiting suckers, and changing harvesting methods.^[^ ^6^ ^]^ In conclusion, various measures have been implemented to reduce nicotine and tar production in tobacco leaves, resulting in a raw material with fewer harmful substances, particularly low tar contents, for cigarette production.

#### 1.2.2 Tobacco Leaf Processing and Cigarette Processing

During cigarette manufacturing, factors such as curing techniques, fermentation, pH, humectants, and additives, can affect nitrosamine formation in tobacco. To reduce the release of harmful components during smoking, researchers have used supercritical CO_2_ technology during tobacco leaf treatment, solvent extraction, and low‐temperature treatment and additives such as combustion‐supporting agents, antioxidants, adsorbents, and catalytic oxidants in cut tobacco. These techniques aim to minimize the release of harmful components and enhance smoking experience.^[^ ^7^ ^]^

#### 1.2.3 Cigarette Filter

Over the last few decades, cigarette manufacturers have developed various filters to minimize exposure to harmful substances. Different types of adsorbent materials have been developed for commercial cigarette filters to remove tar and other harmful particles. Typically, the materials used for making a cigarette filter should be loose, porous, and with good air permeability and filtration efficiency. These materials include sponges; resins; various forms of paper; natural fibers such as cotton, callus, silk, and flax; synthetic fibers such as cellulose esters and ethers; and other functional absorbents such as activated carbon and zeolite.^[^ ^8^ ^]^ Through continuous efforts, cigarette filters can reduce the levels of toxic and carcinogenic substances in cigarette smoke. Studies have revealed that modified filters containing deoxyribonucleic acid (DNA) can significantly reduce PAHs in cigarette smoke without affecting nicotine delivery.^[^ ^9^ ^]^

### 1.3 Materials for Reducing Cigarette Smoke Toxicants

Tobacco with tar levels below 15 mg cigarette^−1^ is internationally recognized to be relatively safe for smokers. However, quitting smoking can result in withdrawal symptoms such as increased negative reactions (e.g., depression, anxiety, annoyance, anger, and insomnia) and decreased positive reactions.^[^ ^10^ ^]^ Therefore, to reduce the harmful effects of tobacco on smokers, it is important to develop adsorption materials that can effectively and selectively lower other toxic substances in cigarette smoke without reducing nicotine content. Nevertheless, the most effective approach to minimize the health hazards of tobacco is to quit smoking. This review aims to provide a systematic summary of adsorption materials, published before 2022, for capturing hazardous compounds from cigarette smoke.

## 2 Toxic Compounds in Cigarette Smoke

**Figure 2:**
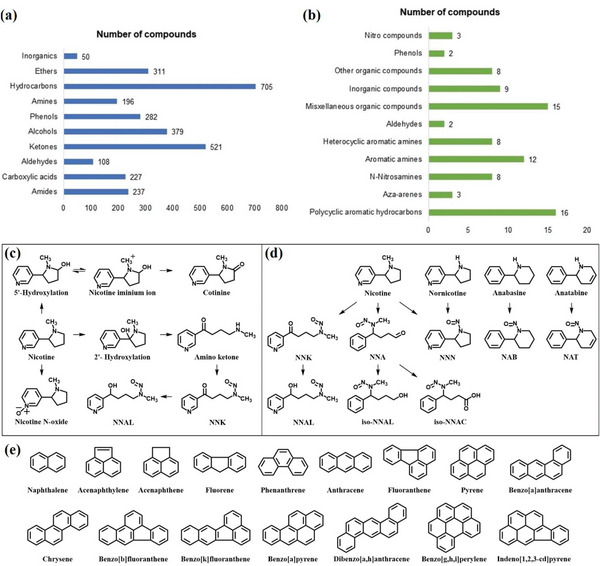
a) Main classes of compounds in tobacco smoke.^[^ ^11^ ^]^ b) 86 carcinogens in cigarette smoke.^[^ ^8a^ ^]^ c) Chemical structure of nicotine, its derivatives, and major pathways of nicotine metabolism.^[^ ^21^, ^22^ ^]^ d) Formation of TSNA through the nitrosation of tobacco alkaloids.^[^ ^24^ ^]^ e) Chemical structures of PAHs in cigarettes.

According to the International Agency for Research on Cancer (IARC), cigarette smoke is a complex mixture of >7000 compounds, of which 50 have been identified as carcinogenic.^[^ ^1a^ ^]^ During tobacco combustion, various gaseous products and suspended particulate matter are produced.^[^ ^11^ ^]^ **Figure** **2**a lists 3016 compounds from the major chemical classes found in tobacco smoke. Since the 1950s, scientific institutions and researchers have conducted several studies on the chemistry of tobacco smoke. In 1997, Hoffmann published a report on the composition of cigarettes and changes in the composition of cigarette smoke, providing a detailed summary of the carcinogens present in cigarette smoke. This review discusses the need to reduce the toxicity and carcinogenicity of cigarette smoke.^[^ ^8a^ ^]^ The “Hoffmann analytes” are recognized by regulators in most countries and regions around the world as relevant to smoking‐related diseases. There are 82 substances in tobacco smoke that are considered harmful. The list of “Hoffmann analytes” includes some PAHs, aromatic amines, N‐nitrosamines, phenols, low boiling point carbonyl compounds, heterocyclic aromatic amines, etc.^[^ ^8^, ^12^ ^]^ Furthermore, Rodgman and Green^[^ ^13^ ^]^ have summarized a more detailed list. The IARC has evaluated the well‐documented risks to humans of 86 carcinogens (Figure 2b) in cigarette smoke.^[^ ^8a^ ^]^

The major ingredients in cigarette smoke vary according to the pyrolysis temperature, atmosphere, and pH of tobacco leaves.^[^ ^14^ ^]^ The popular e‐cigarette is a novel tobacco vapor product that consists of an electrical heating device, an atomizer, and tobacco capsules containing liquid nicotine. During use, tobacco vapor is produced through indirect heating by passing vapor, rather than direct heating of tobacco. The heating temperature in this process is ≈30 °C, which produces a distinctively lower yield of harmful smoke components.^[^ ^15^ ^]^

### 2.1 Pyridine Alkaloids: Nicotine

The major alkaloids in tobacco are nicotine (3‐(1‐methyl‐2‐pyrrolidinyl) pyridine; Figure 2c), nornicotine, anabasine, anatabine, and myosmine, among which nicotine is the primary inducer of tobacco dependence.^[^ ^16^ ^]^ Nicotine is inhaled with smoke and then quickly distributed to human organs, such as the lung, liver, kidneys, and spleen. It easily passes through the membranes and placenta. Nicotine is a naturally occurring alkaloid consumed by tobacco and other plants for hundreds of years and is one of the world's most widely used psychostimulants. Under physiological conditions, nicotine can cross cell membranes and bind stereospecifically to receptors at neuromuscular, adrenal, and autonomic ganglia junctions. It can cross the blood‐brain barrier and is distributed throughout the brain.^[^ ^17^ ^]^ Even short‐term nicotine exposure can cause tachycardia, high blood pressure, nausea, and vomiting. Usually, people resort to e‐cigarettes in response to smoking restrictions, assuming that they would be less harmful to human health, but the nicotine concentration in e‐cigarettes is much higher than that in regular cigarettes, and the risk is still significant.^[^ ^18^ ^]^ Clinical and preclinical evidence suggests that adolescent nicotine exposure increases vulnerability to mood and anxiety disorders later in life.^[^ ^19^ ^]^

Nicotine is rapidly metabolized in the liver (Figure 2c), and roughly 3/4 of nicotine is hydroxylated to 5′‐hydroxy nicotine, mainly by CYP2A6. Then, 5′‐hydroxy nicotine is oxidized to form cotinine by aldehyde oxidase, which is known to relax vascular smooth muscle and dilate blood vessels in vitro. Cotinine is found in much higher concentrations in the blood of tobacco users. Fortunately, most cotinines are metabolized by glucuronidation and excreted through urination.^[^ ^20^ ^]^ However, there exists another metabolic pathway for nicotine. In this pathway, nicotine is hydroxylated to produce 2′‐hydroxynicotine by P450 2A6 and forms an amino ketone NNK (4‐(methylamino)‐1‐(3‐pyridyl)‐1‐butanone) intermediate. NNK is further metabolized into keto acids. Because nitrosation occurs during the chemical synthesis of NNK, it may be a direct cause of nicotine‐induced lung cancer.^[^ ^21^ ^]^ Nitrosation reactions undergo a reaction in which hydrogen in N—H is replaced by NO, forming N—NO. For secondary and tertiary amines, the oxidative cleavage of C—N bonds can occur, yielding pro‐carcinogenic nitrosamines 4‐(methylnitrosamino)‐1‐(3‐pyridyl)‐1‐butanone (NNK), nitrosamino aldehyde (NNA), and N′‐nitrosonornicotine (NNN).^[^ ^16^, ^22^ ^]^

### 2.2 Nitrosamines

Tobacco‐specific carcinogenic nitrosamines (TSNAs) are N‐nitrosamines found exclusively in tobacco products.^[^ ^23^ ^]^ TSNAs are produced in tobacco during both post‐harvest processing and cigarette consumption when alkaloids in tobacco such as nicotine, nornicotine, anabasine, and anatabine, react with nitrosating agents.^[^ ^16^, ^24^ ^]^ Tobacco products contain eight types of TSNA: NNK, NNN, NNA, NAB, NAT, NNAL, iso‐NNAC, and iso‐NNAL (Figure 2d).

TSNA is highly carcinogenic and can lead to various types of malignancies, including those in the oral and nasal cavities, esophagus, lungs, liver, pancreas, and breasts.^[^ ^25^ ^]^ TSNA forms adducts with DNA through metabolic activation of nitrosamines, which causes tissue damage and deterioration.^[^ ^26^ ^]^ This is because, during the metabolism of TSNA, in addition to the formation of DNA adducts, •OH and other reactive oxygen species are also produced, leading to the breakage of single‐stranded DNA and causing DNA damage.^[^ ^16^, ^27^ ^]^ E‐cigarettes and vaping also pose health risks because they contain relatively high concentrations of nicotine and TSNAs. In addition, high levels of TSNAs are detectable in smoking environments, suggesting that non‐smokers are also exposed to the dangers of cigarette smoke owing to secondhand smoke.^[^ ^28^ ^]^

### 2.3 PAHs

PAHs are a class of 2–7 fused aromatic ring compounds containing carbon and hydrogen atoms. PAHs are primarily derived from the incomplete combustion of fossil fuels (e.g., coal, oil, and gas) and other carbon‐containing materials (e.g., wood, paper, and garbage). Low‐temperature combustion, such as that in smoke or forest fires, generates more PAHs than high‐temperature furnace combustion.^[^ ^29^ ^]^ The IARC Monographs Program summarizes the carcinogenicity of 60 individual PAHs.^[^ ^30^ ^]^ Most PAHs are hydrophobic but can pollute water by binding to suspended sediments. Most industrial waste gases and tobacco smoke contain PAHs, which when inhaled, can cause lung and heart diseases.^[^ ^31^ ^]^ Cigarette smoke contains 16 priority toxicant PAHs (Figure 2e and **Table** **1**), including benzo[a]pyrene, which is a potent carcinogen known to increase the risk of various diseases, such as cancer, reproductive diseases, cardiovascular diseases, and obesity.^[^ ^32^ ^]^

**Table 1:** PAH yields of cigarette smoke. Reproduced with permission.^[^ ^31b^ ^]^ Copyright 2006, Elsevier B.V

Name	Molecular weight	Number of rings	Yields [ng cig^−1^]
Naphthalene	128.17	2	2526
Acenaphthylene	152.20	3	613.9
Acenaphthene	154.21	3	319.1
Fluorene	166.22	3	796.0
Phenanthrene	178.23	3	391.3
Anthracene	178.23	3	94.30
Fluoranthene	202.26	4	382.2
Pyrene	202.26	4	242.5
Benzo[a]anthracene	228.29	4	60.51
Chrysene	228.29	4	52.32
Benzo[b]fluoranthene	252.32	5	72.28
Benzo[k]fluoranthene	252.32	5	56.38
Benzo[a]pyrene	252.32	5	63.27
Dibenzo[a,h]anthracene	278.35	5	16.07
Benzo[g,h,i]perylene	276.34	6	274.6
Indeno[1,2,3‐cd]pyrene	276.34	6	48.92

McGrath et al.^[^ ^33^ ^]^ demonstrated that cell wall carbohydrates and lipophilic components in tobacco are primary low‐temperature (≤600 °C) PAH precursors. During the carbonization process, the remaining residual solid (char) undergoes chemical transformation and rearrangement, resulting in the formation of condensed polycyclic aromatic structures between 350–600 °C, which further evolve into PAHs upon heating.

### 2.4 Hydrogen Cyanide

HCN is a highly toxic substance and a representative toxicant found in cigarette smoke. HCN can be absorbed through the mucous membranes and inhibits cytochrome oxidase in the respiratory chain. Its toxic mechanism involves a combination with the Fe^3+^ center in cytochrome oxidase, which blocks the reduction of Fe^3+^ to Fe^2+^ during the oxidation process, leading to the inability of tissue cells in utilizing oxygen and causing hypoxia and asphyxia.^[^ ^34^ ^]^ HCN is extremely toxic with a minimum lethal dose of 30 mg (considering an adult weighing 60 kg as an example) and a maximum allowable concentration of 0.3 mg m^−3^ in air. Mainstream cigarette smoke contains ≈3200 µg of HCN per cigarette. Chronic exposure to HCN can harm the central nervous system; cause amblyopia, retrobulbar neuritis, and sterility; and hinder wound healing in smokers.^[^ ^34a^ ^]^ Therefore, reducing the release of HCN from mainstream cigarette smoke is crucial for protecting the health of smokers.

### 2.5 Carbon Monoxide

CO is a critically hazardous constituent of cigarette smoke that is poisonous and can cause various diseases, including cancer. CO mainly results from the incomplete combustion of coal, automobile exhaust emissions, natural disasters, and the use of stoves. It should be noted that, when smoking, CO is quickly absorbed into the blood, leading to an increase in blood CO content.^[^ ^35^ ^]^ Compared to O_2_, CO has a higher binding affinity for hemoglobin. This binding results in the formation of carboxyhemoglobin (HbCO), which significantly decreases the oxygen‐carrying capacity of hemoglobin, reducing the ability of red blood cells (erythrocytes) to deliver oxygen to tissues.^[^ ^36^ ^]^ The symptoms of CO poisoning include encephalalgia, dizziness, shortness of breath, weakness, retching, and coma. The use of filter tips and cigarette paper can significantly decrease CO emissions from mainstream smoke.^[^ ^37^ ^]^

### 2.6 Formaldehyde

Formaldehyde is classified as carcinogen 1 B and germ cell mutagen category 2 by the European Union.^[^ ^38^ ^]^ When cigarettes are burned, large amounts of saccharide substances in cigarettes are pyrolyzed, producing formaldehyde that exists in both mainstream (yields of ≈20–60 µg) and sidestream (yields of 350–450 µg) smoke.^[^ ^12^, ^14^, ^39^ ^]^ Cigarettes supplemented with sugar continuously increase formaldehyde generation. Notably, the first puff of a cigarette produces unusually high levels of formaldehyde.^[^ ^14b^ ^]^ Studies have indicated that formaldehyde levels in mainstream smoke are affected by factors such as the length of the cigarette rod and smoke accumulation on cigarette filters. Additionally, heat exposure during smoking, during the final puff(s), significantly affects the formaldehyde levels in each puff.^[^ ^40^ ^]^

## 3 Advanced Materials Design for the Adsorption of Toxic Substances in Cigarettes

### 3.1 Cellulose‐Based Materials

#### 3.1.1 Modified Cellulose Acetate

**Figure 3:**
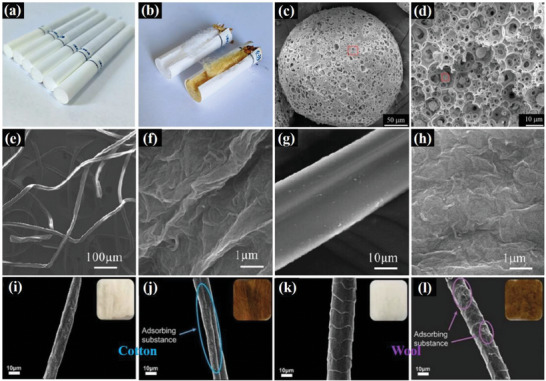
a) Cigarette filters. b) Filters before and after adsorbing cigarette smoke. SEM images of the c) surface structure and d) cross‐section structure of CMC/CA microsphere. e) cellulose acetate filter, f) o‐MWNTs/GO, g,h) o‐MWNTs/GO modified cigarette filter. SEM and photograph of cotton and wool fiber i,j) before and k,l) after filtration. (a‐d) Reproduced with permission.^[^ ^34c^ ^]^ Copyright 2019, MDPI. (e‐h) Reproduced with permission.^[^ ^43^ ^]^ Copyright 2006, Elsevier B.V. (i‐l) Reproduced with permission^[^ ^43^ ^]^ Copyright 2021, Taylor & Francis.

Cigarette filters (**Figure** **3**a) are mainly composed of cellulose acetate, which is obtained by esterifying cellulose molecules with acetic acid. Cellulose acetate fibers (CAF) were first produced on a large scale in the UK in the 1920s, and the Eastman Kodak Company in the US industrialized the production of cellulose acetate cigarette filters in the 1950s. The use of cellulose acetate in cigarette filters presents several advantages, such as no odor, tempered tenacity, oleophobic, high absorption, high resilience, and good thermal stability. Cellulose acetate filters can remove ≈50% of the nicotine and tar from cigarette smoke and some harmful components, such as phenols, while maintaining the flavor.^[^ ^41^ ^]^ Figure 3b shows a comparison of cigarette smoke before and after passing through the filter. However, cellulose acetate has some drawbacks, such as poor high‐temperature thermal stability, low mechanical strength, and easy membrane contamination.^[^ ^42^ ^]^ Consequently, researchers are exploring approaches to design and synthesize cellulose‐modified composite materials to address these issues.

Sun et al.^[^ ^34d^ ^]^ developed a method for preparing functional porous microspheres by synthesizing a composite of carboxymethyl cellulose (CMC) and cellulose acetate (CA) via double emulsion‐solvent evaporation. The researchers also introduced cupric ions, which have a high affinity for HCN, into composite microspheres. The microspheres featured a connected macroporous structure (Figure 3c,d) and were used as adsorbents to reduce HCN by 23.4–50.1% in cigarette smoke. The findings of their study suggest that incorporating functionalized cellulose composites into cigarette filters could enhance their efficacy in reducing the concentration of harmful components in cigarette smoke.

Wang et al.^[^ ^43^ ^]^ developed a modified cigarette filter by immersing a CA filter in a mixture of oxidized multiwalled carbon nanotubes (o‐MWNTs) and graphene oxide (GO), followed by lyophilization (Figure 3e–h). The o‐MWNT/GO composite coating produced in this study considerably enhanced the removal of heavy metals, including Cd and Cr, from mainstream cigarette smoke. This was due to the abundant active adsorption sites and high specific surface area of the lyophilized composite coating on the CA surface of the filter.

Cheng et al.^[^ ^44^ ^]^ developed a CSG aerogel cigarette filter prepared using cellulose nanofibers modified with SiO_2_ nanoparticles and investigated its adsorption properties for toxic substances. Compared to the conventional CAF, the CSG filter exhibited superior adsorption capacity for hazardous substances present in cigarette smoke, with removal rates of 92.2% (tar), 90.3% (total particulate matter, TPM), 95.0% (nicotine), and 20.6% (CO). These results indicate that the CSG filter can effectively reduce the harmful constituents of cigarette smoke.

#### 3.1.2 Modified Natural Cellulose

Natural fibers are widely distributed in nature and are not only abundantly available but also environmentally friendly and inexpensive. Natural fibers have a large specific surface area and internal space, making them potentially suitable for use as adsorbents. Functionalization and modification of natural fiber surfaces are attractive approaches for developing high‐value‐added plant resource products to achieve sustainable goals. Xu et al.^[^ ^45^ ^]^ developed a modified natural cellulose *Juncus effusus* (JE) fiber using polyvinylpyrrolidone (PVP) as a cigarette filter. The PVP‐JE filter tips showed superior filtering and adsorption capabilities for PAHs, with an adsorption rate of 61.8%. This is much higher than that achieved with CA fiber tips (39.2%).

Chen et al.^[^ ^46^ ^]^ investigated the blocking and filtering capacities of cotton (Figure 3i,j) and wool (Figure 3k,l) fibers for PAHs in cigarette smoke. As natural fibers, cotton, and wool are twisted or curled along their longitudinal direction, which increases their flow resistance and enhances their adsorption capacity. The PAHs adsorption efficiencies were 71.0% (cotton) and 60.5% (wool), respectively. Compared with conventional cellulose diacetate cigarette filter tips, filters made of natural fibers demonstrate excellent performance in filtering toxic substances. These findings suggest that natural cellulose fibers are effective, environmentally friendly, cost‐effective sorbents, and provide valuable insights into the design and synthesis of novel cigarette filter tips.

### 3.2 Molecular Sieve

#### 3.2.1 Zeolite Materials

**Figure 4:**
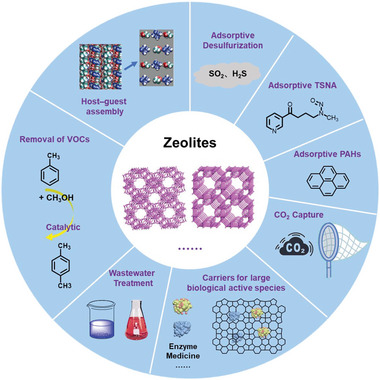
Zeolites are used as sorbents for various applications.

Zeolite is a crystalline aluminosilicate with a 3D framework structure that creates uniformly sized pores with molecular dimensions.^[^ ^47^ ^]^ There are two types of zeolites based on their Si/Al ratios: zeolites with high Si/Al ratios (such as USY, mordenite, and ZSM‐5) and zeolites with low Si/Al ratios (such as NaY and NaA). Owing to their unique shape selectivity and abundant active sites, zeolites have been extensively used as sorbents, catalysts, and carriers. Recently, they have been explored in the life sciences for applications such as slow‐release, enzyme‐mimetic, and antitumor drugs. Zeolites are ubiquitous in daily life and are used for purifying indoor air and softening and decontaminating water, among other applications. One potential application of zeolites is to capture nitrosamines in complex systems (**Figure** **4**).^[^ ^48^ ^]^

Zeolite is a promising adsorption material for toxic tobacco substances because of its unique structure, characterized by ordered micropores and surface adsorption sites. Zeolites can effectively adsorb nitrosamines from mainstream cigarette smoke, and their catalysts can be activated at ≈800 °C during cigarette combustion.^[^ ^49^ ^]^ The electrostatic field in the zeolite channels causes the nitrosamine molecules to be either completely or partially inserted into the channels. The pore size of zeolites provides geometric constraints that can effectively trap carcinogens.^[^ ^50^ ^]^ Moreover, zeolites can selectively adsorb/catalyze volatile nitrosamines with molecular sizes less than or equal to the pore diameter, while bulky nitrosamines that exceed the channel size can be captured and degraded by unique group‐embedded patterns. Some zeolites recognize the functional groups of nitrosamines and nitrobenzenes.^[^ ^51^ ^]^ Therefore, zeolite‐modified filters have the potential to significantly reduce the levels of nicotine, TSNAs, and PAHs in cigarette smoke.

Zhu et al. conducted research on the selective adsorption of TSNAs using zeolites.^[^ ^50^, ^52^ ^]^ Their findings confirmed that zeolites can effectively and selectively adsorb nitrosamines from mainstream cigarette smoke. The adsorption of volatile nitrosamines is affected by several factors such as the temperature, porous structure, and acidity of the adsorbent.

**Figure 5:**
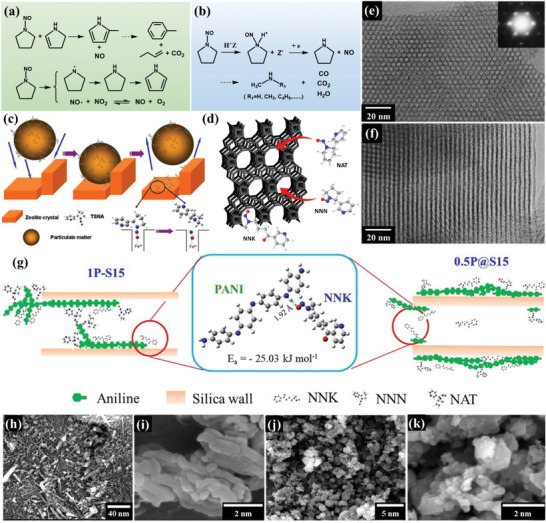
Feasible degradation of N‐nitrosopyrrolidine on a) alkaline zeolites and b) acidic zeolites. Reproduced with permission.^[^ ^54^ ^]^ Copyright 2007, Elsevier Inc. c) Mechanism of removal TSNA from smoke by ferric zeolite. Reproduced with permission.^[^ ^52e^ ^]^ Copyright 2012, Elsevier B.V. d) Possible adsorption model of TSNA by H*β* zeolite. Reproduced with permission.^[^ ^52b^ ^]^ Copyright 2018, Elsevier B.V. TEM images of MCM‐41 along the channel direction e) and perpendicular to it f). Reproduced with permission.^[^ ^58^ ^]^ Copyright 2017, American Chemical Society. g) TSNA adsorption model of PANI/SBA‐15 composites. Reproduced with permission.^[^ ^63c^ ^]^ Copyright 2018, Elsevier B.V. SEM images of h,i) MZ1 and j,k) MZ2. Reproduced with permission.^[^ ^52g^ ^]^ Copyright 2015, MDPI.

a) Temperature: Generally, increasing the temperature leads to a higher airflow velocity, which can hinder the capture of volatile nitrosamines by most zeolites, although, NaZSM‐5 is an exception to this trend.^[^ ^52h^ ^]^
b) Si/Al ratio: Zeolites contain metal ions on their surfaces, and electrostatic fields within their channels. The N—NO group of the nitrosamine molecules carries a negative charge, making it easier for cations in the zeolite to be attracted for adsorption.^[^ ^52h,i^ ^]^ If the zeolite channels are sufficiently wide, the entire adsorbate molecule can be adsorbed. However, for zeolites with narrow channels, only the nitrosamine N—NO group is inserted into the channel while the rest of the molecule remained on the surface.^[^ ^53^ ^]^
c) Acid–base properties: In addition to adsorption, zeolites can catalyze the degradation of nitrosamines. The N—N bond in the N—NO group is the weakest in the nitrosamine structure. The degradation of nitrosamines in zeolites begins with the breakdown of the N—NO bond, releasing NO or NO^+^. In basic zeolites with Na^+^, such as NaZSM‐5 and NaY, the degradation of volatile nitrosamines starts with the cleavage of the N—N bond in the N—NO functional group through a radical reaction process (**Figure** **5**a). On the other hand, in acidic zeolites with proton‐bearing sites such as HZSM‐5 and HY, the degradation of nitrosamines starts with the rupture of the N—N bond, followed by complex reactions between the degradation fragments forming various products such as amines, CO, CO_2_, and H_2_O (Figure 5b). The Brønsted‐Lowry acid sites in acidic zeolites are the main catalytically active sites during the degradation of nitrosamines to form amines. Acidic zeolites are more active than basic zeolites in decomposing nitrosamines because of the presence of protonic bridging hydroxyl sites that form hydrogen‐bonding complexes, allowing for more nitrosamines to be absorbed on acidic zeolites than on their basic counterparts.^[^ ^52^, ^54^ ^]^

The addition of transition metals like copper, cobalt, and ferric species into zeolites can enhance the adsorption and decomposition of nitrosamines, thereby improving their affinity for nitrosamine molecules. However, heavy metal ions such as copper and cobalt are usually toxic, and their loading onto zeolites is not environmentally friendly, limiting their practical application. Ferric zeolite, on the other hand, is a promising alternative for the catalytic removal of TSNAs in smoke. In the zeolite framework, Fe cations have superior catalytic effects on the decomposition of nitrosamines compared to Fe_2_O_3_.^[^ ^52e^ ^]^ The addition of ferric zeolites to cigarette tobacco rods can selectively reduce the TSNA of particulates in smoke by ≈26%. The catalyst remained effective even after two months of storage. Figure 5c shows the mechanism of action of ferric zeolite in removing TSNAs from the particulate matter in smoke. When nitrosamine molecules approach the zeolite pores, the interaction between their N and N groups and the cations in the zeolite leads to instantaneous adsorption. The bulky TSNA molecule inserts its N—NO group into the zeolite cavity, while the thermal movement of the rest of the NNN molecule at elevated temperatures leads to the breakage of the N—N bond.

In 2019, Zhu et al. reported^[^ ^52b^ ^]^ that H*β* zeolite has a high Si/Al ratio, a relatively wide aperture size, and excess hydrions, which make it an excellent adsorbent for TSNAs in solution. The specific aperture size of H*β* is considerable in providing a higher adsorption capacity and selectivity for NNK. NNK had a slender structure that facilitates diffusion inside the zeolite channels (Figure 5d). NNK is more readily adsorbed by H*β* zeolite due to its three adsorptive sites: the N−NO group, the pyridine port, and the carbonyl group. They found that the regenerated H*β* zeolite, after calcination at 873 K, exhibited self‐activation, which increased the TSNA adsorption capacity to 225 µg g^−1^.^[^ ^52c^ ^]^

Typically, the elimination of large PAHs from zeolites is challenging because of the inaccessibility to their micropores. However, zeolites treated with organic salts or surfactants can interact with anions and organic compounds, including PAHs. Recently, an increasing number of zeolites and modified zeolites have been used for PAH adsorption. The sorption mechanism involves dissolving the PAHs in the organic layer of the surfactant and trapping them in the zeolite via various interactions between the zeolite and surfactants that depend on the chemical properties of the PAHs (such as molecular structure, molecular dimension, and dispersive force) and the properties of the zeolites, including their Si/Al ratio, specific surface area, and aperture ratio.^[^ ^55^ ^]^

Researchers also investigated the effects of hydrophobicity,^[^ ^55a^ ^]^ acid–base,^[^ ^55c^ ^]^ and spatial effects^[^ ^55d^ ^]^ on the adsorption of PAHs by zeolite. They found that the adsorption efficiency of PAHs is significantly increased with the *π*—*π* interactions, hydrogen bonding, and hydrophobic effect of functional zeolite. Moreover, the acid‐treated natural zeolite showed the best adsorption of PAHs.The removal rate of some PAHs by the modified zeolite exceeded 85%.^[^ ^55d^ ^]^

#### 3.2.2 Mesoporous Silica Materials

Silicate mesoporous materials are attractive because of their high surface areas and large uniform pores, making them desirable for various applications. The silicate/aluminosilicate mesoporous molecular sieve family M41S includes lamellar (MCM‐50), cubic (MCM‐48), and hexagonal (MCM‐41), which was discovered by the ExxonMobil Corporation in 1992.^[^ ^56^ ^]^ These mesoporous materials have suitable pore sizes (2.0–30.0 nm) and have potential uses as carriers for catalysts, extraction agents, and selective sorbents.^[^ ^57^ ^]^

##### MCM‐41

The hexagonal mesophase MCM‐41 (Figure 5e,f)^[^ ^58^ ^]^ possesses regular and uniform pores and channels with diameters ranging from 15–100 Å. Thus, MCM‐41‐based materials are highly promising for selective adsorption and separation.^[^ ^56^ ^]^ Zhu et al.^[^ ^59^ ^]^ prepared MCM‐41 mesoporous zeolite. The mesoporous structure provides sufficient space for capturing large volumes of nitrosamine (such as NNN), thus improving the adsorption efficiency of NNN. The adsorption capacity of the mesoporous zeolite (MZ25‐12) for NNN is two times higher than that of mesoporous silica (MCM‐41) and over 10 times higher than that of microporous zeolite (ZSM‐5). Subsequently, Zhu et al.^[^ ^60^ ^]^ adjusted the curvature of the MCM‐41 microporous channel. In their study, electrostatic interactions were applied to the TSNA in a solution containing 5% CuO and Al. The composite exhibited excellent performance in various tobacco extract solutions with a TSNA removal rate of 0.31 mg g^−1^.

##### MCM‐48

MCM‐48 features cubic mesoporous structures. Liu et al.^[^ ^61^ ^]^ used cerium to improve catalytic activity and added Ce‐containing MCM‐48 mesoporous material to tobacco or a filter. The results showed that it selectively reduced the PAHs in smoke. Kobylinska^[^ ^62^ ^]^ prepared spherical carbon materials with tunable mesoporous structures by carbonizing polymer materials using MCM‐48 as a template. The surface layer of mesoporous spherical activated carbon material contained proteolytically active carboxyl and hydroxyl groups, and other oxygen‐containing groups. Its adsorption capacity for nicotine (9.2 mmol g^−1^) was better than that of the commercial activated carbon. Moreover, the adsorbent could be reused up to 5–7 times via simple extraction with polar organic solvents.

##### SBA‐15

SBA‐15 is a mesoporous material composed solely of silica. They possess large pores and exhibit thermal, mechanical, and chemical resistance.^[^ ^56^ ^]^ However, SBA‐15 has been found to be inferior to zeolites in the adsorption of volatile nitrosamines due to its inherent defects and lack of metal ions. Nevertheless, the ordered mesopores and large pore sizes of SBA‐15 render the active centers accessible to large feedstock molecules. Thus, modifying these mesoporous siliceous materials has become critical for their application, and various metals, their oxides, and other acidic or basic inorganic and organic functional groups have been introduced to enhance their catalytic properties.

Zhu et al. conducted several modifications on mesoporous SBA‐15 composites to optimize their TSNA adsorption capacity. These modifications include the incorporation of metal oxides such as ZnO, CuO, or Fe_x_O, and the introduction of polyaniline (PANI).^[^ ^63^ ^]^ For instance, ZnO‐modified SBA‐15 composite mesoporous materials have excellent optical and quantum confinement properties and great potential for applications in the field of NNN analysis.^[^ ^63a^ ^]^ CuO/SBA‐15 can trap all NNN in a solution with a concentration of 0.6 mmol because loading CuO on SBA‐15 accelerates the adsorption of carcinogens through the interaction with the N—NO group and causes the degradation of nitrosamines.^[^ ^63b^ ^]^ Additionally, they recycled discarded cigarette filters and treated them with a ferric nitrate solution using SBA‐15. The resulting composite material was then carbonized to form carbon and Fe_x_O particles, which tailored the tortuosity of the mesoporous channels and provided the necessary electrostatic interactions with TSNA in solution. The modified SBA‐15 displayed evident selectivity toward NNK, with a high selectivity of up to 58%.^[^ ^63d^ ^]^ They designed a mesoporous sucker‐type selective sorbent by incorporating PANI into SBA‐15. Under the synergistic action of electrostatic attraction and hydrogen bonding of PANI, the mesoporous adsorbent rapidly captured more TSNA, and its adsorption capacity was even higher than that of activated carbon (Figure 5g).^[^ ^63c^ ^]^

Marcilla et al.^[^ ^64^ ^]^ found that a higher temperature (40 °C), longer reaction time (24 h), and medium stirring rate (700 rpm) during the hydrothermal treatment of SBA‐15 increased the porosity and total pore volume. This resulted in a reduction of 68.1% in TPM, 66.8% in nicotine, and 31.0% in CO. Moreover, the 15/80 SBA‐15 catalyst, synthesized for 15 h at 80 °C, showed a decent reduction effect, with a decrease of 75% in TPM, 66% in nicotine, and 29% in CO.^[^ ^64a^ ^]^

##### Micro/Mesoporous Composites

While zeolites are limited in their ability to trap bulky nitrosamines due to their micropore size, SBA‐15 has shown higher activity in the degradation of bulky TSNA, such as NNN, compared to zeolite NaY. To overcome the lack of geometric constraints of SBA‐15, researchers have synthesized composite materials with hierarchical porous structures to enhance their adsorption activity for volatile nitrosamines in cigarette smoke.

Zhu et al.^[^ ^52g^ ^]^ synthesized two hierarchical composites, MZ1 (Figure 5h,i) and MZ2 (Figure 5j,k), by introducing HZSM‐5 or NaY zeolites into mesoporous silica SBA‐15 or MCM‐41, respectively. The prepared composites exhibited significantly improved adsorption capacities for TSNA and other gaseous substances by combining the advantages of each material. MZ2 was found to trap ≈30% of carcinogens from cigarette smoke. Wu et al.^[^ ^65^ ^]^ prepared sandwich‐type micro/mesoporous Si/O/C/N composite aerogels and placed them on cigarette filters. The removal efficiency of the carbonyl compounds in cigarette smoke by the composite filter was 13.2–75.9%. Notably, the crotonaldehyde removal rate was 75.9%.

#### 3.2.3 Aluminophosphate Molecular Sieves

Aluminophosphate molecular sieves, such as AlPO_4‐n_, are widely used as catalyst supports and physical adsorbents owing to their electrically neutral frameworks, diverse pore structures, large surface areas, and thermal stability, making them important functional materials. However, when the substitution of metals in aluminophosphates breaks the electroneutrality of the framework, resulting in the formation of MeAPO‐n, which exhibits chemical adsorption properties.

Liu et al.^[^ ^66^ ^]^ investigated the cigarette smoke adsorption activity of a cobalt‐substituted aluminophosphate, CoAPO‐11, which is mesoporous. However, because of the highly crystalline AEL topology and the presence of Co in the skeleton, which provides both acidic and basic sites, CoAPO‐11 has the potential to effectively adsorb phenol, HCN, and crotonaldehyde. The adsorption behavior of phenol on CoAPO‐11 was found to conform to a pseudo‐second‐order kinetic model. Adsorption was performed using film and intraparticle diffusion.

### 3.3 Carbon Materials

#### 3.3.1 Activated Carbon and Bio‐based Carbon Materials

**Figure 6:**
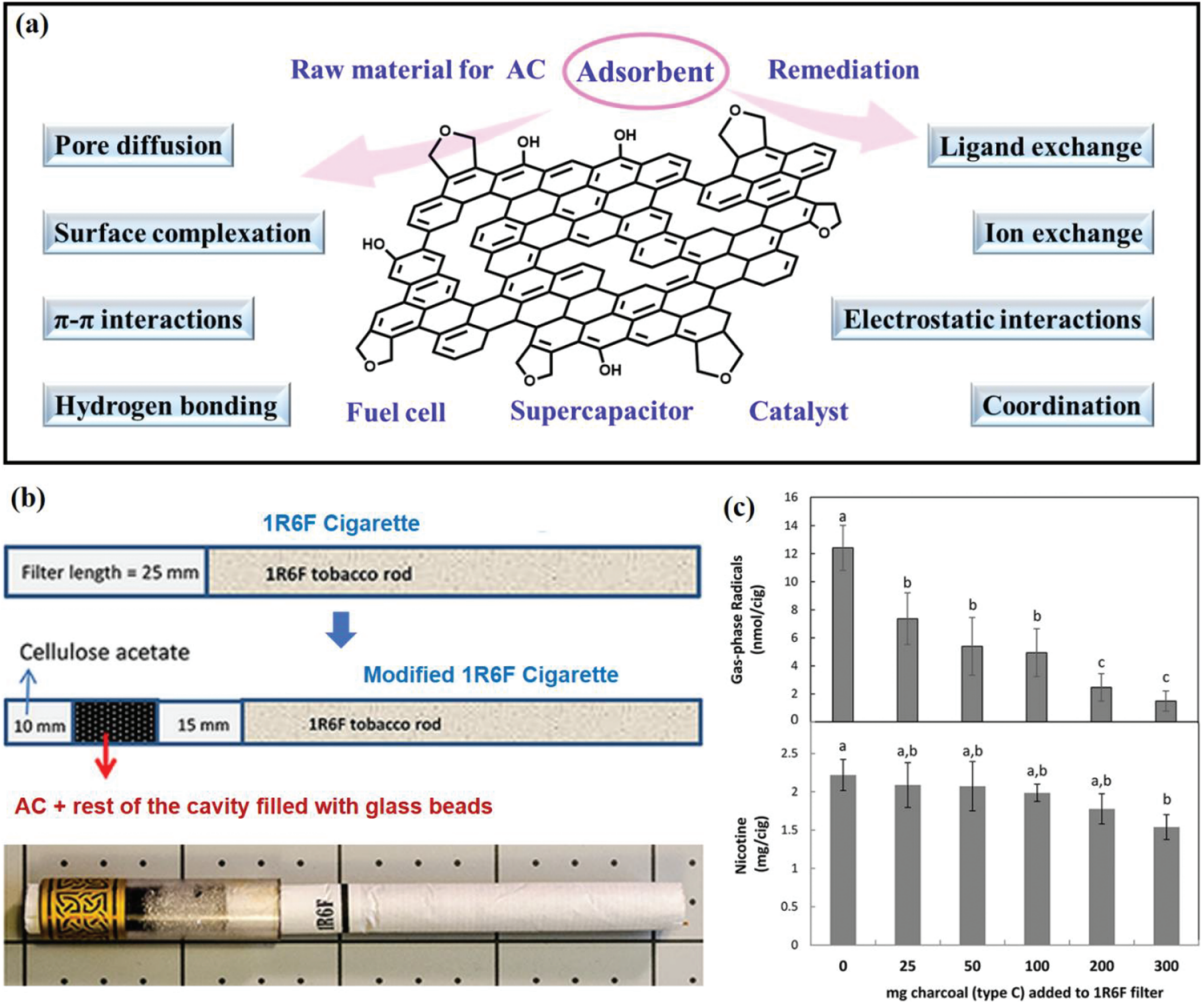
a) Schematic of the application and the adsorption mechanisms of biochar. b) Process diagram of adding AC to make composite filter1R6F and c) adsorption capacity of free radicals and nicotine by the 1R6F filter. Reproduced with permission.^[^ ^74^ ^]^ Copyright 2018, American Chemical Society.

Activated carbon (AC) is a widely used adsorbent with a large specific surface area, abundant surface‐active groups, and stable physical properties. Due to these features, AC is an excellent candidate for environmental protection applications. It is a porous and inert material commonly used as a carrier (**Figure** **6**a).^[^ ^67^ ^]^ Biochar is a type of AC derived from biomass. It possesses the greatest advantages of AC and is useful for various applications such as contaminant sorbents, soil amendments, carbon sequestration, catalysts, and energy storage materials.^[^ ^68^ ^]^ It has surface functional groups such as carboxyl, phenolic hydroxyl, and acid anhydride, which contribute to its adsorption characteristics and ability to strongly adsorb Pb^2+^,^[^ ^69^ ^]^ Hg^0^,^[^ ^70^ ^]^ SO_2_,^[^ ^71^ ^]^ CO_2_, CH_4_, H_2_S,^[^ ^72^ ^]^ and plant nutrients (N, P, and K). Depending on the desired environmental application, biochar can be modified using different techniques, including PH regulation, oxidation, and gasification.^[^ ^73^ ^]^ These modifications can enhance its adsorption capacity, selectivity, and other properties, making it an even more useful material for environmental protection.

In 1954, American Tobacco launched the first AC filter cigarette, which had a charcoal flavor and negatively affected cigarette sales. However, Richie et al.^[^ ^74^ ^]^ later reported that adding AC to filters could remove free radicals and nicotine from mainstream smoke. Nowadays, commercially available cigarettes contain 45–180 mg of charcoal (Figure 6b,c). Research has demonstrated that increasing the quantity of AC (25–300 mg) in filters reduces the gas‐phase radicals (41–88%) and nicotine content (6–31%).

In recent years, researchers have been focusing on studying modified AC owing to its potential to adsorb the harmful components of cigarette smoke. While AC is conducive to the adsorption of TSNA in solution owing to its high porosity, its hydrophobic properties limit its adsorption capacity for TSNA and other nonpolar organic compounds. To enhance AC's affinity for the TSNA, researchers have been exploring modifications such as loading metal oxides like ZnO, CuO, Al_2_O_3_, and Fe_2_O_3_ onto porous AC.^[^ ^75^ ^]^

**Figure 7:**
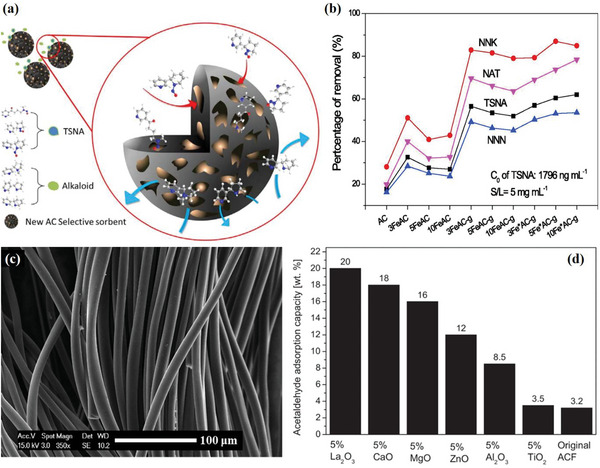
a) Schematic of the selective adsorption of TSNA by a novel zeolite‐like adsorbent prepared from ferric‐modified coconut shell AC and b) AC and its modified analogs at 303 K. Reproduced with permission.^[^ ^76^ ^]^ Copyright 2018, Elsevier B.V. c) SEM image of activated carbon fibers ACF‐K‐20 (specific surface area ∼2000 m^2^ g^−1^); acetaldehyde adsorption capacity of d) metal oxide modified ACF‐K‐20. Reproduced with permission.^[^ ^83^ ^]^ Copyright 2015, Elsevier B.V.

Coconut‐shell‐based AC is known for its low cost, high mechanical stability, high purity, dust‐free nature, and optimal porosity, making it an advantageous adsorbent. It possesses a wide range of aperture sizes, ranging from micropores to macropores, which makes it appropriate for adsorption, similar to zeolites. However, like other ACs, coconut shell AC lacks adsorption selectivity. Therefore, modifying agents must be introduced to adjust surface morphology and create an optimal microenvironment for the selective capture of hazardous compounds.^[^ ^52f^ ^]^ The research shows that metal cations are effective in inducing N—NO groups via electrostatic interactions with the negatively charged functional group N—NO to modify AC and enhance the capture of TSNA. Researchers have coated AC with ZnO via wet impregnation, resulting in a sample that removed 80% of the TSNA.^[^ ^75^ ^]^ To influence the pore structure for TSNA adsorption, AC was modified by grinding to adjust the shapes of its pores and cracks. Ferric acetate or iron nitrate was then carbonized to form numerous carbon nanoparticles on the AC, altering the surface tortuosity of the fissures and pore walls (**Figure** **7**a,b).^[^ ^76^ ^]^ This sorbent was able to capture 62% of TSNA in a 1796 ng/mL tobacco extract solution, exhibiting a high capacity of 222 µg g^−1^.

Yuliusman et al.^[^ ^77^ ^]^ investigated the use of AC derived from corn stalks, which they modified with 0.5% NiO using KOH activation at 750 °C. The resulting NiO‐modified AC exhibited high selectivity for adsorbing harmful components of cigarette smoke, with 82.16% selectivity, as compared to 29.9% for CO. This study identified the KOH‐750 AC sample as the best quality carbon source for modification, highlighting the potential of agricultural waste‐derived AC for environmental and health applications.

Every year, trillions of cigarette butt litter are dumped worldwide, causing environmental pollution and posing a major challenge to disposal regulations.^[^ ^78^ ^]^ To address this issue, Zhu et al.^[^ ^79^ ^]^ used discarded cigarette filters as carbon precursors, and low‐cost metal nitrates as additives to prepare a hierarchical carbonic structure that selectively captures TSNA. The metal nitrate underwent thermal degradation and was converted to a metal oxide, providing indispensable electrostatic attraction to the negatively charged N—NO functional groups. Furthermore, they modified used cigarette butt litter with Al(NO_3_)_3_ and Fe(Ac)_2_ and prepared a modified sorbent with well‐developed porosity (350 m^2^ g^−1^), and well‐exposed metal oxides. This sorbent adsorbed 41% of the TSNA in a tobacco extract solution and trapped Pb^2+^ with improved capacity(153 mg g^−1^).^[^ ^7b^ ^]^

Besides AC, other natural and organic materials have also been extensively studied for their ability to reduce toxicants in tobacco smoke due to their affordability and environmentally friendly properties. China and the USA alone generate ≈1.4 billion tons of agricultural biomass waste annually. Waste management principles demand minimizing and recycling waste, recovering energy, and properly disposing of waste.^[^ ^80^ ^]^ Therefore, developing biochar materials with specific adsorption properties for toxic compounds in cigarette smoke is a promising area of research that could benefit both environmental sustainability and economic growth.

#### 3.3.2 AC Fiber

Activated carbon fibers (ACFs) are fibrous adsorption materials prepared by carbonizing and activating organic fibers at high temperatures. In comparison to grained AC, they exhibit excellent adsorption performance owing to their larger surface area, smaller aperture size, larger adsorption capacity, and faster adsorption kinetics. ACFs have a wide range of applications in gases, organic solvents, supercapacitors, and fuel cells.

ACFs possess several desirable properties such as good acid and base adaptability, electrical conductivity, chemical stability, and recyclability, making them ideal environmentally friendly materials. Additionally, ACFs exhibit natural hydrophobicity, with a lower content of oxygen groups on the surface (735–865 µmol g^−1^) compared to most ACs (1570–4289 µmol g^−1^).^[^ ^81^ ^]^ Owing to their specific structure, ACFs have a higher affinity for nonpolar and weakly polar molecules, whereas polar molecules are not as easily adsorbed. For instance, ACFs have greater adsorption capacity for nonpolar toluene than ACs, which have higher specific surface areas.^[^ ^82^ ^]^ Moreover, the maximum adsorption capacity of polar acetaldehyde can reach 20%, which is lower than that of nonpolar benzene (31%) and toluene (53%).^[^ ^81^, ^83^ ^]^

Baur et al.^[^ ^83^ ^]^ developed a highly efficient adsorbent by loading basic metal oxide nanoparticles (NPs) onto ACF (Figure 7c) for the specific adsorption of acetaldehyde (Figure 7d). Compared to unmodified ACF, NPs deposition resulted in a 10‐fold increase in the amount of acetaldehyde adsorbed. In this study, three types of surface adsorption sites: physisorption on the surface carbon (containing oxygen) and two types of chemisorption on the MgO surface were investigated. Wu et al.^[^ ^84^ ^]^ incorporated berberine‐loaded ACF into a CA filter to create a modified B‐ACF cigarette filter. The cigarette filter effectively reduced the HCN content in cigarette smoke and mitigated the inhibitory effect of cigarette smoke on oral peroxidase activity. Cigarette technology can incorporate low‐toxicity plant ingredients into filter materials to reduce toxicity.

However, the practical industrial application of ACF is restricted by the high cost of fiber precursors and associated processing costs.^[^ ^85^ ^]^

#### 3.3.3 Carbon Nanotubes

**Figure 8:**
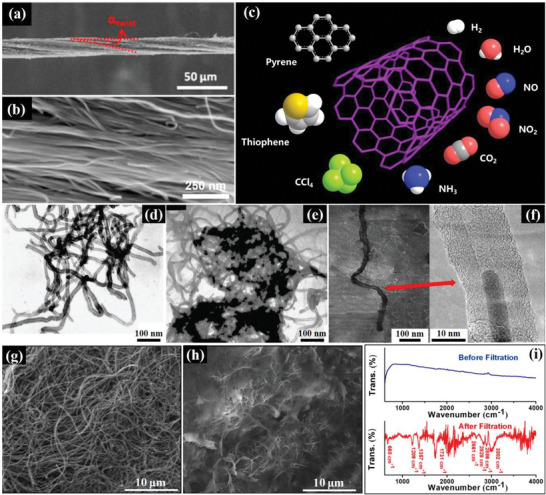
SEM images of a) CNT fibers after densification showing significant torsion (*α_twist_* = 10°) and b) arrangements in the CNT fiber after densification. Reproduced with permission.^[^ ^97^ ^]^ Copyright 2017, Elsevier Ltd. c) Schematic representation of SWCNTs and their applications. d) TEM image of O‐CNTs, e) TEM, and f) HRTEM image of O‐CNTs after adsorption of smoke. Reproduced with permission.^[^ ^94^ ^]^ Copyright 2005, Elsevier B.V. SEM images of MWCNTs before g) and after h) adsorption of smoke, i) FITR spectra of MWCNTs before and after adsorption of smoke. Reproduced with permission.^[^ ^96^ ^]^ Copyright 2020, American Chemical Society.

Carbon nanotubes (CNT) are a type of crystalline carbon material discovered by Iijima et al. in 1991. They are cylindrical tubes made of carbon with diameters on the nanometer scale (**Figure** **8**a,b). CNTs are formed by rolling graphite sheets, resulting in a continuous, unbreakable hexagonal mesh structure with carbon atoms at the corners of each hexagon. The cylindrical structure is composed of one or more layers of graphite sheets curled in the axial direction, with both ends closed by a hemispherical cap.

CNTs possess unique electromagnetic, mechanical, optical, and thermal properties, making them useful in various applications, such as hydrogen storage materials, composite enhancers, catalyst supports, and superconducting materials. Owing to their unique surface properties and aperture structures,^[^ ^86^ ^]^ they are also promising adsorbents (Figure 8c) for gases such as CO_2_,^[^ ^87^ ^]^ NO_x_,^[^ ^88^ ^]^ H_2_,^[^ ^89^ ^]^ NH_3_,^[^ ^90^ ^]^ and VOC.^[^ ^91^ ^]^ In addition, CNTs exhibit an intense adsorption affinity for organics, including n‐nonane, CCl_4_, and thiophene,^[^ ^92^ ^]^ with typically higher adsorption capacities than AC and other carbon adsorbents.

CNTs have been reportedly used for the adsorption of harmful components from mainstream smoke.^[^ ^93^ ^]^ Oxidized carbon nanotubes (O‐CNTs) can be used to adsorb molecules from mainstream smoke because of their curved structures with aggregated pores (Figure 8d,e). Phase transitions and capillary condensation occurred when the smoke hits the walls of the carbon nanotubes, allowing the O‐CNTs to adsorb nicotine and tar in the hollow core and between layers (Figure 8f).^[^ ^94^ ^]^ Zhou et al.^[^ ^95^ ^]^ synthesized a carbon nanotube mixture (CNTM) by catalyzing polypropylene and organically modifying montmorillonite nanocomposites with Ni_2_O_3_ to remove toxic substances from smoke. CNTM showed excellent adsorption performance owing to the relatively large pore volume, geometric restrictions provided by the presence of CNTs, and strong chemical adsorption capacity for benzo[a]pyrene or phenols, which was owing to *π*—*π* interaction between the aromatic modules. Pandey et al.^[^ ^96^ ^]^ inserted an MWCNTs membrane (Figure 8g) into a cigarette filter and prepared a composite filter that could effectively absorb PM2.5, with a removal rate of up to 99%. It also removed other harmful substances (≈30%) from cigarette smoke (Figure 8h,i).

Although CNTs show potential as adsorbents for cigarette smoke, it is important to note that the aggregation of CNTs remains a challenge to their widespread application. To overcome these shortcomings, carbon nanotubes can be dispersed in a solution by surface oxidation and surfactant coating.

#### 3.3.4 Graphene

**Figure 9:**
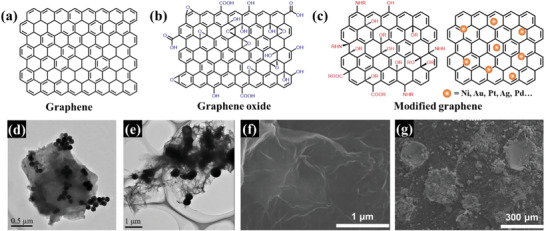
Chemical structure of a) graphene, b) GO, and c) modified graphene. TEM images of (d) graphene/Fe_3_O_4_ (Fe_3_O_4_ diameter is 80 nm) and e) graphene/Fe_3_O_4_@SiO_2_ composites. Reproduced with permission.^[^ ^100^ ^]^ Copyright 2019, WILEY‐VCH Verlag GmbH & Co. KGaA, Weinheim. f) SEM image of graphene aerogel. Reproduced under the terms of the CC–BY Creative Commons Attribution 3.0 International License.^[^ ^103^ ^]^ Copyright 2018, published under licence by IOP Publishing. g) SEM image of ACS/GO. Reproduced with permission.^[^ ^98b^ ^]^ Copyright 2020, Elsevier B.V.

The structures of graphene and its derivatives are shown in **Figure** **9**a–c. The carbon atoms in graphene are densely packed in a honeycomb lattice and exhibit unique electronic, chemical, and mechanical properties. The unique structure of graphene gives it excellent physical and chemical properties, making it a promising material for functional adsorption. GO has a surface inlaid with abundant oxygen‐containing functional groups (Figure 9b). Therefore, the presence of abundant active sites makes it suitable for further surface chemical modification, while its negative charges enable it to interact with positively charged substances, making it a good adsorbent. Furthermore, because of its dense array of atoms, GO can significantly increase dispersion forces in composite materials, which is conducive to the adsorption of small molecules.^[^ ^98^ ^]^ Recently, graphene and its derivatives (Figure 9a–c) have become popular nanomaterials for effectively adsorbing pollutants from water, such as heavy metal ions, inorganic anions, oils, dyes, and other organic compounds (Figure 9d,e).^[^ ^98^, ^99^ ^]^ While most investigations have focused on aqueous pollutants, some studies have reported the adsorptive removal of hazardous substances from the gas phase,^[^ ^98c^ ^]^ particularly hazardous substances from cigarette smoke (Figure 9d,e).^[^ ^100^ ^]^ Graphene mainly relies on *π*—*π* interaction to adsorb NNK.^[^ ^101^ ^]^

Zhu et al.^[^ ^102^ ^]^ discovered that the shape selectivity of NNK on graphene‐based materials in aqueous solutions of TSNA was due to the difference in electronic interactions, rather than the geometric confinement of the pores. This electronic interaction‐based selectivity showed a higher adsorption capacity and faster adsorption rate for TSNA and was less limited by diffusion and mass transfer. Xu et al.^[^ ^103^ ^]^ showed that graphene aerogels (Figure 9f) could spontaneously and exothermically adsorb NNK from aqueous solutions, and higher‐pH solutions favored NNK adsorption on graphene aerogels. The hexagonal cells of graphene aerogel and pyridine ring in NNK molecules jointly promoted *π*—*π* dispersion interaction between them. Zhang et al.^[^ ^98b^ ^]^ blended GO and aminated chitosan (ACS) to synthesize ACS/GO composite material (surface area 7.460 m2 g^−1^) (Figure 9g) and found that GO was more dispersed. The enhanced aniline adsorption performance of ACS can be attributed to the addition of GO, as GO contains abundant functional groups such as carboxyl and phenolic hydroxyl groups. These functional groups can form hydrogen bonds or undergo acid–base reactions with aniline, leading to improved adsorption. Additionally, the presence of *π* bonds allowed further reactions between GO and aniline.

Graphene‐based composite materials are receiving research attention and are expected to receive increasing focus in the future due to their outstanding adsorption performances.

### 3.4 Novel Porous Materials

#### 3.4.1 MOF Adsorption Materials

**Figure 10:**
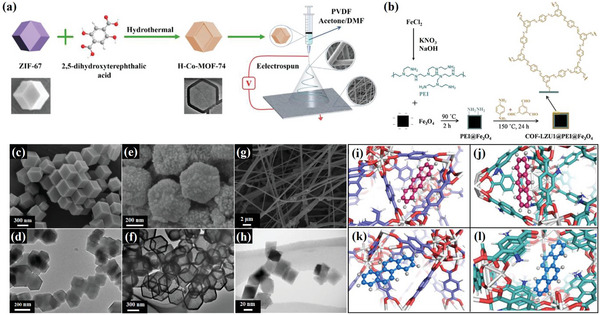
a) Preparation process of hollow Co‐MOF‐74 incorporated nanofiber membranes (H‐NFMs). Reproduced with permission.^[^ ^106^ ^]^ Copyright 2022, Elsevier B.V. b) Schematic of preparation of COF‐LZU1@PEI@Fe_3_O_4_. Reproduced with permission.^[^ ^110a^ ^]^ Copyright 2017, Springer‐Verlag GmbH Austria. SEM and TEM images of c,d) ZIF‐67 and e,f) H‐Co‐MOF‐74. g) SEM image of H‐NFMs.^[^ ^106^ ^]^ h) TEM image of COF‐LZU1@PEI@Fe_3_O_4_.^[^ ^110a^ ^]^ Molecular structure of i) UiO‐66(ANT), j) UiO‐66(CRY), k) NH_2_‐UiO‐66(ANT), and l) NH_2_‐UiO‐66(CRY). Reproduced with permission.^[^ ^105b^ ^]^ Copyright 2020, Elsevier Ltd.

Metal–organic frameworks (MOFs) are a type of porous material composed of organic ligands and metal ions through self‐assembly, forming a highly crystalline and periodic 3D network structure. Because of the various coordination numbers of different metal ions and the complexity of their coordination with organic ligands, MOFs exhibit a wide range of multidimensional skeleton structures.^[^ ^104^ ^]^ Compared to other porous materials such as AC and zeolites, MOFs have several advantages, including a large specific surface area, adjustable pore size, micro‐and mesoporous structures, diversity in pore size and skeleton structure, and tunable pore surface functional groups with unsaturated metal coordination sites. Furthermore, MOFs have high adsorption capacities for many toxic gases at room temperature and atmospheric pressure, providing a competitive advantage for PAHs adsorption. Furthermore, MOFs have the potential to selectively adsorb PAHs because of their superior porosity and strong *π*—*π* stacking effects on aromatic hydrocarbons.^[^ ^105^ ^]^ Zango et al.^[^ ^105b^ ^]^ conducted molecular docking studies to investigate the adsorption of PAHs on MOFs and determine the optimal host‐guest binding interaction and receptor‐binding sites that form the most stable conformation. As shown in **Figure** **10**i–l, anthracene (ANT) molecules preferentially bind to the outer pores of the MOFs, whereas chrysene (CRY) molecules prefer the inner center of the pore.

The modification of MOFs into hollow or multishell structures has been explored by some researchers, leading to improved adsorption performance. In particular, hollow MOFs offer advantages over conventional MOFs owing to their large internal cavities and thin shells. The unique structure of MOFs reduces the diffusion length during the recognition and adsorption processes. Huang et al.^[^ ^106^ ^]^ demonstrated the preparation (Figure 10a,c–g) of hollow Co‐MOF‐74 incorporated nanofiber membranes (H‐NFMs) and their application for PAHs adsorption. The results showed that the H‐NFMs increased the PAH removal rate to >75%. As researchers continue to develop new MOFs with different metal ions and organic ligands, the study of their toxic gas adsorption properties will progress.

Chen et al.^[^ ^107^ ^]^ synthesized Ce‐BDC MOFs to scavenge harmful hydroxyl radicals (•OH) in cigarettes by combining Ce ions with terephthalic acid (TA) (CE‐BDC) and then self‐assembling them under the template effect of soluble starch. The formation of Ce—O coordination bonds and oxygen vacancies in Ce‐BDC MOFs had the ability to convert Ce^3+^ to Ce^4+^, and eliminate >90% of •OH. On the other hand, Yang et al.^[^ ^108^ ^]^ prepared MOF@CF filter materials for nicotine adsorption by combining MOFs ((ZIF‐8, HKUST‐1, MOF‐74, UiO‐66, UiO‐66—NH_2_, and UiO‐66—OH)) with bamboo‐derived cellulose fiber. The UiO‐66@CF exhibited the best mechanical properties and good recyclability, while also achieving a nicotine adsorption efficiency of 90%. The XPS results indicated that all MOF@CFs had open metal sites that bound to nicotine except for ZIF‐8@CF. DFT calculations proved that the highest occupied molecular orbital and lowest unoccupied molecular orbital energy difference of UiO‐66 was 4.9 eV, which was the closest to that of nicotine, making it more favorable for nicotine adsorption.

MOFs are considered one of the most promising adsorption materials for harmful substances in tobacco because of their tunable pore structures and exceptional physicochemical properties. However, some limitations hinder their practical application, such as weak dispersion forces arising from a large amount of void space and a lack of open metal sites. Additionally, the commercial application of MOFs for adsorbing harmful substances in cigarette smoke is limited owing to the high cost of MOFs materials.

#### 3.4.2 Covalent Organic Framework Adsorption Materials

Covalent organic frameworks (COFs) are a type of highly porous materials that consist entirely of organic compounds that are renowned for their exceptionally lightweight properties. Unlike 2D COFs, 3D COFs have a distinct framework that results in a higher specific surface area and a lower crystal density, which makes them more versatile. One of their most significant advantages is their exceptional ability to adsorb gases adsorption capacity, which makes them ideal for storing gases, such as H_2_, CO_2_, CH_4_, and others.^[^ ^109^ ^]^ Additionally, magnetic COFs are highly effective adsorbents for enriching and detecting PAHs in PM2.5, water, and soil.^[^ ^110^ ^]^

Chen et al.^[^ ^110a^ ^]^ developed a COF‐based magnetic adsorbent (Figure 10b,h) to extract PAHs from the solid phase by attaching COF‐LZU1 to magnetic nanoparticles functionalized with polyethyleneimine (COF‐LZU1@PEI@Fe_3_O_4_), which proved to be highly effective for the extraction of PAHs. The excellent performance of this adsorbent was mainly due to strong *π*—*π* stacking as well as hydrophobic interactions. Zhu et al.^[^ ^110d^ ^]^ synthesized a clover‐shaped nanotitania‐functionalized COFs (CSTF‐COF) for the dispersed solid‐phase extraction of N‐nitrosamines in water. It showed excellent extraction ability to N‐nitrosamines through hydrophobic, *π*—*π* superposition, and hydrogen‐bonding interactions.

#### 3.4.3 Molecularly Imprinted Polymers

**Figure 11:**
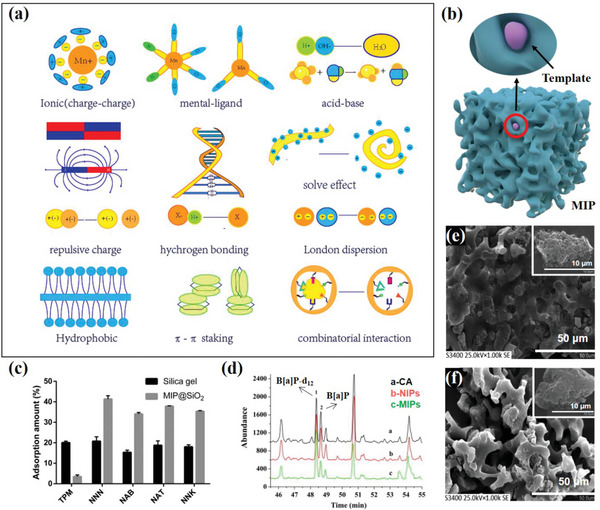
a) Different types of interaction for functional materials are designed for specific recognition and imprinting. b) Schematic of the three‐dimensional structure of MIPs. c) Adsorption amount of silica gel and MIP@SiO_2_ to TPM and TSNA in mainstream cigarette smoke. Reproduced with permission.^[^ ^118^ ^]^ Copyright 2015, WILEY‐VCH Verlag GmbH & Co. KGaA, Weinheim. d) Ion chromatogram of the mainstream smoke of various filtered cigarettes. Reproduced with permission.^[^ ^120^ ^]^ Copyright 2013, Elsevier Ltd. e) SEM picture of MIM (nicotine surface‐imprinted monolith) and f) non‐imprinted monolith. Reproduced with permission.^[^ ^119^ ^]^ Copyright 2018, Elsevier B.V.

Although the materials discussed above have been used as adsorbents, they are generally not selective and tend to adsorb non‐specifically. Thus, it is imperative to develop adsorption materials with high selectivity, particularly for the harmful components in cigarette smoke. To achieve this, adsorption materials can be designed to take advantage of different types of intermolecular forces at the molecular level. One particularly interesting area in supramolecular chemistry is the development of molecularly imprinted polymers (MIPs). MIPs are functional materials with high affinity and selectivity for target molecules. Molecular recognition interactions rely on various non‐covalent bond forces, including electrostatic interaction, hydrogen bond, coordination bond between metal atoms and ligands, hydrophobic interaction, *π*—*π* stacking, charge‐dipole interaction, van der Waals force, and more (**Figure** **11**a).^[^ ^111^ ^]^ The effectiveness of molecular recognition depends on the synergy, direction, and selectivity of these forces.

Molecular imprinting can be divided into three steps: recognition and binding, cross‐linked polymerization, template removal, and rebinding. Finally, three‐dimensional cavities were created that matched the spatial structure of the template molecule and included functional groups that could bind specifically to the template molecule.^[^ ^112^ ^]^ Figure 11b depicts the possible three‐dimensional structure of the MIPs.

One of the most significant applications of MIPs is as sorbents for solid‐phase extraction. MIPs have been explored for the adsorption of harmful components, such as nicotine and PAHs, in cigarette smoke. In these studies, the harmful components were used as template molecules to prepare the MIPs for use as adsorbents in solid‐phase extraction. In 2004, Knopp et al.^[^ ^113^ ^]^ first synthesized MIPs using B[a]P as the template molecule. The study indicated that the polarity of the functional monomer and crosslinking agents are critical in determining the affinity and retention behaviors and that the cross‐linker is more critical than the monomer. Baggiani et al.^[^ ^114^ ^]^ synthesized pyrene‐imprinted microbeads with molecular recognition capabilities for PAHs and demonstrated that the molecular recognition of PAHs by MIPs is controlled by the shape and dimensions of the binding sites through hydrophobic interactions. Msagati et al.^[^ ^115^ ^]^ prepared a composite of reduced GO and Pyr‐imprinted polymer that could successfully extract PAHs from water samples as a solid phase. Xu et al.^[^ ^116^ ^]^ used NNK as a template molecule to synthesize hollow molecularly imprinted polymer microspheres that showed excellent selectivity, fast mass transfer rate, and efficient adsorption performance, with the adsorption capacity being three times that of the hollow non‐imprinted polymer to N‐nitroamine. Xie et al.^[^ ^117^ ^]^ synthesized surface MIPs (SMIPs) with SiO_2_ nanospheres bearing vinyl functional groups that specifically recognize nicotine. The results showed that the SMIPs efficiently and selectively adsorbed nicotine.

Numerous studies have investigated the use of MIPs as cigarette filter materials. Zhu et al.^[^ ^118^ ^]^ grafted MIPs with nicotinamide as a template onto the surface of silica gel, obtaining MIP@SiO_2_, which was used as a filter additive at the interface between the filter tip and tobacco rod to absorb TSNA in mainstream cigarette smoke. The results showed that MIP@SiO_2_ selectively adsorbed TSNA without simultaneously adsorbing the total PM (Figure 11c), thus reducing TSNA in mainstream cigarette smoke without altering the taste of cigarettes. Li et al.^[^ ^119^ ^]^ prepared a surface‐imprinted monolith with a specific recognition ability for nicotine (Figure 11e,f), and analyzed the adsorption performance of the prepared materials. The results showed that MIM selectively adsorbed nicotine in tobacco smokers with an adsorption rate of up to 99.4%. Chen et al.^[^ ^120^ ^]^ synthesized a pyrene‐imprinted polymer as part of a cigarette filter tip. The MIPs showed good affinity toward pyrene in mainstream cigarette smoke (Figure 11d), with a binding capacity of 18.3 mg g^−1^. After replacing the CA filter with the MIPs‐filter, the PAH content in cigarette smoke was reduced by 36.4%.

**Figure 12:**
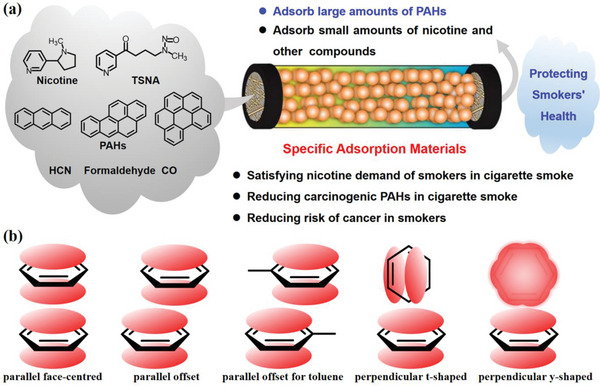
a) Design of specific adsorption materials for PAHs in tobacco. b) Possible aromatic stacking arrangements.^[^ ^121^ ^]^

PAHs are the primary carcinogen found in cigarette smoke. Therefore, it is crucial to develop advanced materials with excellent selective adsorption properties for PAHs (**Figure** **12**a). However, there are few reports on the effective adsorption of PAHs from tobacco smoke using MIP. This could be due to PAHs having very few functional groups, which makes it difficult for MIPs to effectively recognize them through strong molecular interactions. Instead, they rely on only weak *π*—*π* stacking interactions, which are not strong enough. Figure 12b shows possible aromatic stacking arrangements that researchers can use to design selective adsorption materials to capture PAHs.^[^ ^121^ ^]^

## 4 Conclusion and Perspective

In this review, we highlighted the latest advancements in the development of advanced materials aimed at reducing the toxic compounds in cigarette smoke.

Cigarette smoke contains over 7000 substances, of which >250 are harmful to human health, with at least 50 being carcinogenic. The main harmful components of cigarette smoke are nicotine, TSNA, PAHs, HCN, CO, and formaldehyde. Nicotine is the main addictive chemical component in cigarettes. It is a carcinogenic precursor that can be converted into TSNA during catabolic processes. Eight TSNA, namely NNK, NNN, NNA, NAB, NAT, NNAL, iso‐NNAC, and iso‐NNAL, were identified in tobacco products. Cigarette smoke contains 16 priority toxicant PAHs, particularly benzo[a]pyrene, which are potent carcinogens that increase the risk of lung and heart diseases.

This review summarizes various adsorption materials that can be used to reduce the harmful components in cigarette smoke, including cellulose‐based materials, zeolites, mesoporous silica, carbon materials, MOFs, COFs, and MIPs. We also discussed the adsorption efficiency and possible mechanism of these adsorption materials in reducing harmful components in cigarette smoke. Modified CA, modified natural cellulose, and carbon materials (AC, CNTs, and graphene) can absorb nicotine, PAHs, HCN, CO, and other substances from cigarette smoke. Molecular sieves can reduce the levels of nicotine, TSNAs, and PAHs in cigarette smoke and have a specific adsorption capacity for TSNAs. MOFs, COFs. Notably, MIPs have the potential to selectively adsorb PAHs due to their superior selectivity and strong *π*—*π* stacking interactions on aromatics, but the high cost limits their practical applications. These materials can be used to prepare filter materials for removing harmful substances in cigarette smoke.

While significant progress has been made, challenges remain in the development of advanced materials that can effectively reduce the toxic substances in cigarette smoke without significantly affecting smoking satisfaction. It is important to point out that nicotine is an addictive substance in cigarette smoke. If nicotine is removed and reduced indiscriminately along with the well‐known carcinogenic substances, PAHs, smokers may end up smoking more cigarettes to satisfy the addictive demand of nicotine receptors. Therefore, it is desirable to develop advanced materials with specific adsorption behavior and high selectivity for future research. Ideally, the desired materials would adsorb a small amount of nicotine or even zero uptakes in cigarette smoke while having high specificity and strong recognition and adsorption for carcinogenic PAHs. In summary, researchers will continue to explore and develop advanced materials with large adsorption capacities, excellent selectivities, and low costs to minimize the harmful effects of cigarette smoke on human health.

## Conflict of Interest

The authors declare no conflict of interest.
